# Australian R U OK?Day campaign: improving helping beliefs, intentions and behaviours

**DOI:** 10.1186/s13033-019-0317-4

**Published:** 2019-09-14

**Authors:** Anna M. Ross, Bridget Bassilios

**Affiliations:** Centre for Mental Health, Melbourne School of Global and Population Health, The University of Melbourne, Melbourne, VIC Australia

**Keywords:** R U OK, Suicide prevention, Public awareness campaign, Helping intentions, Helping behaviour, Helping beliefs

## Abstract

**Background:**

Suicide is a major public health concern and has been recognised as a public health priority. R U OK?Day aims to prevent suicide by encouraging and empowering Australians to reach out to friends and family who might be experiencing personal difficulties. This study aims to update the evaluation of the public awareness campaign ‘R U OK?Day’ that was conducted using 2014 data.

**Methods:**

Data from 2013 participants were collected via an online survey following the R U OK?Day campaign implemented in 2017. Outcome measures included campaign awareness and participation, past 12-month help-seeking, helping beliefs, helping intentions and helping behaviours. Data were analysed using z-tests, Chi square and regression analyses in SPSS.

**Results:**

Both campaign awareness and participation have increased since 2014, from 66% and 19% to 78% and 32%. Campaign exposure was associated with stronger beliefs in the importance and the ease of asking “Are you okay?”, and increased the likelihood of intentions to use recommended helping actions by two to three times compared to those not exposed to the campaign. Participants who were exposed to the R U OK?Day campaign were up to six times more likely to reach out to someone who might be experiencing personal difficulties compared to those not exposed to the campaign. Interestingly, those who had sought help from a mental health professional in the past 12 months were more likely to be aware of, and participate in, the campaign, suggesting people experiencing mental health issues recognise the value of seeking—and giving—social support.

**Conclusions:**

The R U OK?Day campaign continues to be relevant and effective in spreading key messages about the importance of reaching out to others and empowering members of the community to have conversations about life problems. The campaign’s impact is increasing over time through increased campaign awareness and participation, and improving helping beliefs, intentions and behaviours. Ongoing monitoring and evaluation of the campaign’s impact is vital and may inform potential changes needed to further enhance its impact.

## Background

Suicide is a major public health concern and its prevention has been recognised as a public health priority globally with close to 800,000 people dying by suicide every year [1]. In 2017, in Australia alone, 3128 people died by suicide, which is a 9.1% increase from 2016 [2]. Preventing suicide has also been identified as a priority in Australia’s Fifth National Mental Health and Suicide Prevention Plan [3].

### Preventing suicide in the community

Public health campaigns contribute to suicide prevention by creating community understanding and awareness, and a willingness to engage in action that can support other focused and specialist suicide prevention initiatives [1]. Therefore, the World Health Organisation (WHO) requires that an awareness component is included in national suicide prevention strategies [1].

Campaigns that aim to increase awareness of suicide prevention on a community level by improving both knowledge about suicide prevention and attitudes towards help-seeking have been found to be mostly successful. Reviews of the effectiveness of some suicide awareness campaigns and suicide prevention campaigns using mass media [4–6] have shown improved knowledge and awareness of suicide [7, 8], improved attitudes towards suicide prevention [9, 10], and increased calls to suicide prevention helplines [11–13]. Although causality is unconfirmed, decreases in suicide rates and attempts have also been observed following campaigns that were adequately powered [14–16] with longer-term campaigns that achieve support from the community found to be the most likely to reduce suicide rates [6].

However, campaign evaluation findings have been mixed. Some studies have found campaigns to have both positive influences or no effects on attitudes towards preventing suicide and help-seeking intentions [4, 5], while one study found reduced positive attitudes towards help seeking [17]. The longer-term impact of such campaigns is mostly unknown due to the lack of long-term follow-up [4]. Further, some campaigns have not had the intended impact on help-seeking and suicide rates [18] and others have possibly had unintended negative effects of reducing positive attitudes towards help-seeking in certain sub-populations (e.g., adolescents experiencing depressive symptoms [17, 19]; Japanese people living in highly-populated areas [20]).

### Social support in preventing suicide

The majority of the suicide prevention campaigns reviewed by Pirkis et al. [5], Fountoulakis et al. [6], and Dumenesil and Verger [4] focus on improving knowledge and attitudes towards suicide prevention and encouraging help seeking from a mental health professional or a helpline for suicidal thoughts. They also tend not to specifically focus on providing education about supporting someone who may be experiencing personal difficulties. With social isolation identified as a risk factor for suicide, and social support and relationships found to be protective against suicide [21, 22], communities can play a critical role in preventing suicide [1].

Support and intervention from social networks can be particularly useful, as those experiencing suicidal ideation have been found to have more negative attitudes towards help-seeking and lower help-seeking intentions [23]. However, while friends and family are often best positioned to notice warning signs, they can be unsure of these and how to effectively intervene [24, 25]. Further, the preference to disclose suicidal thoughts to family and friends increases the protective value of social networks and emphasises the need to upskill individuals in providing support to others who might be at risk of suicide [26, 27].

Given the valuable role social relationships play in protecting against suicide, providing community education on reaching out to others who may be at risk of suicide is an ideal opportunity for intervention. Through interventions that aim to improve social connectedness, communities can provide social support to vulnerable individuals, reduce stigmatising attitudes towards talking about suicidal thoughts, break down barriers to help-seeking, and promote resources and support [28]. The literature has emphasised the importance of systematic evaluation and longer-term follow up on the impact of suicide prevention awareness campaigns [4, 29, 30].

### Suicide prevention campaign R U OK?Day

R U OK?Day is a significant Australian public health promotion campaign that is held each year in September and encourages utilising social support in dealing with life events and personal difficulties. Launched in 2009 and ultimately aiming to prevent suicide in the community, the campaign is consistent with aspects of the Interpersonal Theory of Suicide aiming to prevent suicide by increasing social support and a sense of belonging [21]. Specifically, R U OK? empowers the community to connect with, and support each other in dealing with life events and difficulties by initiating helping conversations [31, 32]. The R U OK? Campaign encourages the use of a four-step model to have these conversations: (1) ask the person how they’re going, (2) listen without judgement, (3) encourage the person to take action, such as seeking support from a mental health professional and (4) check in with the person by following up with them at a later time. Community, school or workplace events, such as morning teas or guest speaker presentations, also take place on R U OK?Day, with an increasing number of ongoing campaign promotions occurring throughout the year (e.g., the Conversation Convoy which is a national road trip involving visits to numerous regional and remote Australian communities to spread the R U OK? message, online resources and social media communications).

An initial evaluation of the impact of R U OK?Day was conducted in 2014 through an Australia-wide population survey [32]. With a total sample size of 2000 participants, the evaluation found that awareness of the R U OK?Day campaign had increased, with two-thirds of participants found to be aware of the R U OK? Campaign and 1 in 5 participating in R U OK?Day. Most participants who were aware of the campaign reported positive perceptions of the campaign and its impact on people’s willingness to talk to others about their problems and to seek professional help. However, the campaign’s impact on helping intentions, helping behaviours and confidence in reaching out to someone when they indicate they are ‘not OK’ were not reported on in the published findings of the 2014 evaluation [32].

Using data collected by R U OK? in 2017, this study aims to independently update the evaluation of the R U OK?Day campaign and to determine its impact on helping attitudes and behaviours. Specifically, we aim to answer the following research questions:

1. Has campaign awareness and participation in 2017 increased since 2014?
2. Has campaign awareness and participation in each age group in 2017 increased since 2014?
3. Is there a difference in campaign awareness between those who sought help from a mental health professional in the past 12 months and those who did not?
4. Does campaign exposure predict intentions to help a close friend who might be, or is obviously, experiencing personal difficulties?
5. Does campaign exposure predict belief in the importance and ease of asking a close friend who appears to be experiencing personal difficulties about what’s troubling them?
6. Does campaign exposure predict the likelihood of reaching out to someone who might be experiencing personal difficulties?

## Method

### Data sources

We obtained human research ethics approval for this project from The University of Melbourne (HREC# 1852632.1). We utilised pre-existing data that are routinely collected by R U OK? Limited through its subcontractor The Online Research Unit. The data were collected via online cross-sectional surveys following the 2017 R U OK?Day campaign with quota sampling used to ensure representation of Australian residents aged 16 years and over across age, gender and geographic location.

Participants were recruited via online research panels. Potential participants had previously agreed to be contacted about participating in online surveys and were sent an email from the subcontractor inviting them to take part. The survey was administered to participants via a secure online platform, with no R U OK? stimulus (such as branding or logos) shown to participants and was expected to take 15 min to complete. Participants were rewarded with points for completing the survey which earnt each participant $2 of value in redeemable gift cards. The survey requested demographic and mental health information including help seeking from a mental health professional; awareness of, and participation in, the R U OK?Day campaign; helping intentions, beliefs and behaviours; and perceptions regarding brand credibility and perceived impact of the R U OK?Day campaign. The data collection methods, survey questions and scoring methods were consistent with 2014, with some additional questions added to the 2017 survey to assess helping beliefs, helping intentions, and reaching out to others (helping behaviours).

*Demographic information* used for our purposes included gender, age group, and geographic location.

*Help seeking* from a mental health professional for participants’ own mental health concerns was measured using a single question: “In the past 12 months or so have you seen a counsellor, doctor or psychologist because of a mental health problem?” with three response options: yes (1), no (0), or would rather not say (2).

*Campaign awareness* was measured in 2 ways. First, participants were provided a list of 17 health advocacy and wellbeing support organisations (one of which was R U OK?) and asked to select those they had heard of. Second, participants were directly asked ‘Have you heard of R U OK?Day?’ with their responses coded as yes or no. Participants were considered to be aware of the campaign if they indicated they had heard of R U OK? in either the free recall question or the direct question, in which case they received a score of 1; those who were not aware received a score of zero.

*Campaign participation* was measured by asking participants “Did you do anything or participate in any activities as part of R U OK?Day on Thursday September 14 this year?”. Only participants who had indicated that they were aware of R U OK?Day were asked about their participation in the campaign. Responses were classified into 2 categories to determine the total participation score: yes (1), no and unsure (0). Participants who responded affirmatively to having participated in R U OK?Day were further asked to report what they did to participate from a list of 11 common campaign participation activities:

1. Asked someone face-to-face if they were OK
2. Telephoned someone to ask if they were OK.
3. Messaged someone online to ask if they were OK (e.g., Facebook, Twitter, Instagram, Snapchat).
4. Emailed someone to ask if they were OK.
5. SMS messaged someone to ask if they were OK.
6. Posted a general comment about R U OK?Day on social media (e.g., Facebook, Twitter, Instagram, Snapchat).
7. Looked into options for myself with regards to professional help.
8. Looked into options for someone else with regards to professional help.
9. Attended an event or activity associated with R U OK?Day.
10. Helped to organise an event or activity associated with R U OK?Day.
11. Spent extra time with family, friends and/or others.

*Campaign exposure* was created by the researchers as an overall exposure to the R U OK?Day campaign variable that combined campaign awareness and campaign participation scores. Scores ranged from 0—not aware, 1—aware but did not participate, to 2—aware and participated, with higher scores indicating higher campaign exposure.

*Intentions* to initiate a conversation with someone was determined by asking participants what they would most likely do in two different scenarios to help a close friend experiencing personal difficulties. The first scenario involved a close friend who was *obviously* very troubled about something, and the second scenario asked about a close friend who *might be* troubled about something that was thought not to be serious. Participants indicated what they would most likely do in each scenario by selecting one response from the following list of actions for each scenario:

1. Nothing—just carry on as if everything was normal.
2. Do something to distract them from their problem.
3. Try and leave as soon as possible without it being obvious.
4. Spend more time with them than planned.
5. Ask them if they are alright.
6. Try and cheer them up by making jokes.
7. Ask them to talk about what was troubling them.
8. Something else (Please specify).
9. None of these.

We classified responses into two categories: “recommended actions” (responses 4, 5 and 7, coded as ‘1’) and “non-recommended actions” (responses 1, 2, 3, 6 and 9, coded as ‘0’) consistent with campaign messaging and the prior research on helping actions [33–36]. Where Response 8 ‘something else’ had been selected and the alternate action specified, the actions provided were also classified into these categories by the researchers. Response 9 ‘none of these’ was coded as “non-recommended actions”.

*Helping beliefs* refer to beliefs about the importance and ease of asking a friend who appeared to be troubled about their wellbeing. This was measured with two questions. The first question—“If a friend appeared troubled how strongly would you feel you should either ask or not ask about what’s troubling them?”—required participants to indicate their response on an 11-point likert-scale, from 0 ‘Definitely feel I should ask’ to 10 ‘definitely feel I shouldn’t ask’, with 5 ‘unsure’ indicating a neutral response. The second question—“Please rate how easy or difficult you would personally find it to ask a friend about their wellbeing if they appeared troubled.”—also required participants to indicate their response on an 11-point likert scale, from 0 ‘Very easy’ to 10 ‘Very difficult’ with 5 ‘unsure’ similarly indicating a neutral response. Based on these rating scales, lower scores indicate stronger helping beliefs. However, for the purposes of analysis and to improve the intuitiveness of the findings, these scores were reversed so that higher scores indicate stronger helping beliefs.

*Reaching out (helping behaviour)* was measured by the frequency with which participants had reached out to offer support to others. Participants were provided a list of supportive behaviours and asked to indicate the frequency with which they had engaged in these behaviours in the past month. The list of supportive behaviours included:

1. Asked someone face-to-face if something was troubling them.
2. Telephoned someone to ask if something was troubling them.
3. Messaged someone online to ask if something was troubling them (e.g., Facebook, Twitter, Instagram, Snapchat).
4. Emailed someone to ask if something was troubling them.
5. SMS messaged someone to ask if something was troubling them.
6. Listened to someone talk about their problems.
7. Contacted a support service on behalf of someone.
8. Referred someone who is troubled to a support service.

Participants selected from four frequency categories to capture their estimate of how often they had engaged in each behaviour over the last month: ‘I haven’t done this’, ‘1–2 times’, ‘3–5 times’, or ‘more than 5 times’. Scores for ‘reaching out’ (helping behaviour) were classified into binary categories: 1—‘yes reached out to someone’ if one or more supportive behaviours was endorsed and 0—‘no did not reach out’ if no supportive behaviours were endorsed.

### Data analysis

The data were analysed using SPSS version 23 and MedCalc online calculator. Data were weighted by age, gender, and state/territory to ensure that the sample was representative of the Australian general population. The population statistics were sourced from the Australian Bureau of Statistics’ most recent Estimated Resident Population release which was based on the 2016 Census data.

Frequencies and percentages for participant demographic variables, awareness and participation were computed and compared to 2014 data using two-proportion one-tailed z-tests. Differences in awareness between those who sought help from a mental health professional and those who did not were calculated using chi-square analyses. Cramer’s V was calculated to indicate effect size of the chi-square analyses due to multiple groups being compared, which is interpreted the same as a correlation [37].

Separate logistic regressions were conducted to predict helping intentions and reaching out to someone who appeared troubled (helping behaviour). Predictor variables included campaign awareness, campaign participation, past help seeking from a professional, sex, age group and geographical location. A linear regression was used to predict helping beliefs using these same predictor variables. The Enter method was used for all regression models. Assumption testing for the regression models included collinearity diagnostic statistics in SPSS, as well as Box-Tidwell testing for the logistic regressions and Q–Q plots for linear regression revealed the assumptions for the logistic and linear regressions were met.

## Results

A total of 2013 participants completed the post-campaign survey in 2017. Participant demographic details are presented in Table 1, with similar sized demographic groups demonstrating that both samples were weighted similarly to ensure representativeness of the Australian population.

**Table 1:** Demographic variables for survey respondents in 2017 compared to 2014 and 2016 census data

	2014^a^ (N = 2000) weighted		2017 (N = 2013) unweighted		2017 (N = 2013) weighted		2016 census data^b^
	n	% of total sample	n	% of total sample	Weighted n	Weighted %	% of total Australian population
Gender							
Female	1013	50.6	1039	51.6	1021	50.7	50.7
Male	987	49.4	974	48.4	992	49.3	49.3
Age group							
16–24	330	16.5	219	10.9	300	14.9	15.7 (15–24)
25–34	342	17.1	366	18.2	372	18.5	17.7
35–44	353	17.7	381	18.9	337	16.7	16.5
45–54	340	17.0	367	18.2	329	16.3	16.3
55–64	288	14.4	307	15.3	291	14.5	14.4
65+	347	17.3	373	18.5	384	19.1	19.3
Geographical location							
Metropolitan	1280	64.0	1429	71.0	1542	76.6	67.0
Non-metropolitan	720	36.0	584	29.0	471	23.4	33.0

^a^2014 data reported in Mok et al. [32]

^b^Census data from Australian Bureau of Statistics [38, 39]

### Awareness of, and participation in, the R U OK?Day campaign

Awareness and participation percentages are presented by demographic variables in Table 2. Compared to 2014, both awareness and participation have significantly increased overall, as well as across all sex and geographic groups, and most age groups (except the 16–24 age group where the increase in awareness was not statistically significant). The 16–24 age group have remained the most aware age group, and the 25–34 age group are still most likely to participate in activities related to the campaign. Campaign awareness amongst the 45–54 age group increased substantially by 16%, being the fourth most aware age group in 2014 and the second most aware age group in 2017. Similar to the 2014 findings, in 2017 the 65+ age group had the lowest awareness and participation rates. However, since 2014, these rates have increased significantly and are now comparable to those of the age groups over 45 years. In the 2014 evaluation, there were significant age differences in participation rates, ranging from 8% (in the 65+ age group) to 29% (in the 25–34 age group), 21% difference [15.6–26.8%], *χ*^2^ (1) = 51.87, Cramer’s V = .161, *p *< .001, however these differences appear to be less pronounced in 2017, across a smaller range of 25–41%, 17% difference [10.1–23.3%], *χ*^2^ (1) = 24.18, Cramer’s V = .110, *p *< .001.

**Table 2:** Weighted awareness of R U OK?Day and participation in R U OK?Day by sex, age and geographical location in 2017 compared to 2014

	Awareness						Participation					
	Participants in 2014^a^ (N = 2000)		Participants in 2017 (N = 2013)		Percent difference [95% CI]	*p* value	Participants in 2014^a^ (N = 2000)		Participants in 2017 (N = 2013)		Percent difference [95% CI]	*p*-value
	n	%	n	%	%		n	%	n	%	%	
Gender												
Female	731	72.2	847	82.9	10.7 [7.1–14.3]	< .001	144	19.7	268	31.6	11.9 [8.1–15.6]	< .001
Male	582	59.0	715	72.1	13.1 [8.9–17.2]	< .001	106	18.2	226	31.6	13.4 [9.6–17.1]	< .001
Age group												
16–24	255	77.3	250	83.2	5.9 [–.04–12.0]	.028	67	26.3	93	37.3	11.0 [3.7–18.2]	.001
25–34	232	67.8	282	75.9	12.8 [6.1–19.4]	.009	67	28.9	117	41.3	12.4 [5.4–19.2]	< .001
35–44	225	63.7	258	76.5	12.8 [6.0–19.5]	< .001	33	14.7	78	30.4	15.7 [9.5–21.8]	< .001
45–54	222	65.3	267	81.1	15.8 [9.2–22.3]	< .001	35	15.8	75	28.2	12.4 [6.1–18.6]	< .001
55–64	196	68.1	220	75.5	7.4 [0.1–14.6]	.022	34	17.3	61	27.7	10.4 [3.6–17.1]	.002
65+	183	52.7	286	74.5	21.8 [14.9–28.5]	< .001	14	7.7	70	24.5	16.8 [11.6–21.9]	< .001
Geographical location												
Metropolitan	830	64.8	1179	76.4	11.6 [8.2–15.0]	< .001	167	20.1	385	32.7	12.6 [9.4–15.8]	< .001
Non-metropolitan	483	67.1	383	81.4	14.3 [9.3–19.1]	< .001	83	17.2	110	28.6	11.4 [6.5–16.4]	< .001
Total	1313	65.7	1562	77.6	11.9 [9.1–14.7]	< .001	250	19.0	494	31.6	12.6 [9.93–15.25]	< .001

^a^2014 data reported in Mok et al. [32]

The increase in campaign awareness from 2014 to 2017 was found to be significantly different between age groups, *χ*^2^ (5) = 11.80, Cramer’s V = .077, *p *= .038, ranging from 6% (in 16–24 age group) to 22% (in 65+ age group). The increase in campaign participation also differed between age groups, *χ*^2^ (5) = 26.07, Cramer’s V = .129, *p *< .001, ranging from 11% (in the 16–24 age group) to 17% (in the 65+ age group).

### Campaign awareness and participation and help-seeking

Participants who indicated they had sought help from a mental health professional in the last 12 months were more likely to be aware of and participate in *R U OK?Day*, *χ*^2^ (2) = 35.90, Cramer’s V = .134, *p *< .001, and *χ*^2^ (2) = 46.37, Cramer’s V = .172, *p *< .001 respectively.

Weighted descriptive data for the other dependent variables from the 2017 survey are presented in Table 3 by campaign exposure levels.

**Table 3:** Weighted descriptive data for dependent variables from the 2017 survey by campaign exposure levels: not aware of the campaign; aware of the campaign but did not partcipate; aware of and participated in the campaign

Variable	2017 participants (N = 2013) n (%)	Not aware (N = 451) n (%)	Aware (N = 1562) n (%)	Aware and participated (N = 494) n (%)
12-month help-seeking from a mental health professional				
Sought help	331 (16.4)	38 (8.5)	152 (14.2)	141 (28.5)
Didn’t seek help	1616 (80.3)	387 (85.8)	890 (83.3)	339 (68.6)
Rather not say	66 (3.3)	26 (5.7)	26 (2.5)	14 (2.8)
Intentions to help a close friend who might be troubled				
Intend to help	1700 (84.4)	337 (74.7)	921 (86.2)	442 (10.7)
No action intended	314 (15.6)	114 (25.3)	147 (13.8)	53 (89.3)
Intentions to help close friend who is obviously troubled				
Recommended action	1854 (92.1)	384 (85.3)	1012 (94.8)	458 (92.6)
Non-recommended action	159 (7.9)	66 (14.6)	56 (5.2)	37 (7.4)
Reaching out/helping offered in past month				
Help offered	1601 (79.5)	309 (68.5)	824 (77.1)	469 (94.8)
No help offered	412 (20.5)	142 (31.5)	244 (22.9)	26 (5.2)

	M (SD)	M (SD)	M (SD)	M (SD)
Belief in importance of asking	5.91 (3.30)	5.45 (3.11)	6.05 (3.25)	6.05 (3.54)
Belief in ease of asking	6.03 (2.76)	5.53 (2.65)	5.96 (2.78)	6.64 (2.71)

### Campaign exposure predicting helping intentions and helping behaviour

Results from the binary logistic regressions predicting intentions to help a close friend who might be or obviously appeared troubled, and reaching out to someone who might be troubled (helping behaviour) using recommended helping actions are presented in Table 4.

**Table 4:** Logistic regression analyses predicting helping intentions and helping behaviour in 2017

Predictor variable	Intentions to help close friend who might be troubled		Intentions to help close friend who is obviously troubled		Helping behaviour/reaching out to someone who might be troubled	
	B (SE)	Odds ratio [95% CI]	B (SE)	Odds ratio [95% CI]	B (SE)	Odds ratio [95% CI]
Sex						
Male (ref)						
Female	.335 (.129)**	1.398 [1.086–1.799]	.527 (.177)**	1.694 [1.198–2.397]	.783 (.121)***	2.189 [1.727–2.774]
Age group						
16–24 (ref)						
25–34	− .382 (.211)	.683 [.451–1.033]	− 3.20 (.271)	.726 [.427–1.236]	− .271 (.256)	.763 [.462–1.259]
35–44	− .574 (.210)**	.563 [.373–.850]	− .591 (.267)*	.554 [.328–.935]	− .916 (.239)***	.400 [.250–.640]
45–54	.052 (.230)	1.054 [.672–1.653]	.217 (.310)	1.242 [.676–2.281]	− .791 (.243)**	.454 [.282–.731]
55–64	.662 (.268)*	1.939 [1.146–3.282]	1.152 (.412)**	3.166 [1.412–7.099]	− 1.004 (.244)***	.366 [.227–.591]
65+	.607 (.247)*	1.834 [1.131–2.976]	1.229 (.389)**	3.417 [1.594–7.328]	− 1.332 (.231)***	.264 [.168–.415]
Geographical location						
Non-metropolitan (ref)						
Metropolitan	− .119 (.163)	.888 [.645–1.220]	− .022 (.225)**	.979 [.630–1.519]	− .041 (.140)	.960 [.730–1.262]
Exposure to campaign						
No exposure (ref)						
Some exposure (aware but did not participate)	.700 (.146)***	2.013 [1.514–2.678]	1.092 (1.99)***	2.979 [2.017–4.400]	.297 (.131)**	1.346 [1.040–1.741]
Highest exposure (aware and participated)	1.077 (.190)***	2.936 [2.022–4.262]	.836 (.231)***	2.308 [1.467–3.631]	1.863 (.232)***	6.445 [4.094–10.146]
Help seeking						
No help sought (ref)						
Sought help	− .022 (.179)	.978 [.689–1.390]	− .160 (.232)	.852 [.541–1.343]	.632 (.209)**	1.881 [1.250–2.831]
Rather not say	− .374 (.302)	.688 [.381–1.243]	− .584 (.356)	.558 [.278–1.121]	− .103 (.330)	.902 [.473–1.722]
Constant	1.074 (.248)***	2.926	1.515 (.325)***	4.549	1.303 (253)***	3.679
Model *χ*^2^	105.114*** (df = 11)		103.934*** (df = 11)		236.968*** (df = 11)	

* *p* < .05, **p < .01, ***p < .001

Both intentions to help a close friend who *might be* troubled about something and intentions to help a close friend who is *obviously* troubled using recommend actions were significantly predicted by sex, age groups and campaign exposure. Specifically, females, people aged between 35 and 44 and 65+ years, and people exposed to the campaign were more likely to have stronger intentions to help using recommended actions. Campaign exposure was the strongest predictor of intentions to help using recommended actions, those with highest exposure being almost three times more likely to have intentions to help a close friend who might be troubled using recommended actions, OR = 2.94 [2.02–4.26], *p* < .001, and more than twice as likely to have intentions to help a friend who was obviously troubled using recommended actions, OR = 2.31 [1.47–3.63], *p* < .001, compared to those who were not exposed to the campaign. Seeking professional mental health support in the past 12 months was not a significant predictor of helping intentions.

Reaching out to someone who might be troubled about something (helping behaviour) was significantly predicted by campaign exposure, sex, age group and past help seeking. Campaign exposure was again the strongest predictor variable, with those who reported highest exposure being up to six times more likely to reach out to someone who might be troubled compared to those who were not aware, OR = 6.45 [4.09–10.15], *p* < .001. Specifically, females, people not in the 25–34 age group, and people who had sought professional help in the past 12 months were most likely to offer help.

### Campaign exposure’s impact on helping beliefs

Predictors of beliefs about the importance of asking a friend who appeared troubled about what’s troubling them and the ease of asking about their wellbeing are presented in Table 5. Beliefs about asking someone who might be troubled if they are OK were significantly predicted by campaign exposure, sex, age group and geographical location. Specifically, an increased likelihood of having stronger helping beliefs was associated with being female, in an older age-group and from a non-metropolitan area; and having had more exposure to the campaign. Sex was the strongest predictor of both belief variables, with females more likely to have stronger beliefs in the importance and ease of asking R U OK?, unstandardised *b* = .70 (.15), *p* < .001 and unstandardised *b* = .81 (.12), *p* < .001. However, the models explained very little variance in both outcomes.

**Table 5:** Linear regression analyses predicting beliefs and ease in asking someone who appeared troubled ‘R U OK?’

	Belief in importance of asking		Belief in ease of asking	
	Unstandardised *b* coefficient [95% CI]	p-value	unstandardised *b* Coefficient [95% CI]	*p*-value
Sex	.679 [.393, .965]	< .001	.811 [.575, 1.047]	< .001
Age group	.198 [.112, .284]	< .001	.167 [.096, .237]	< .001
Geographical location	− .450 [− .791, − .108]	.010	− .383 [− .664, − .101]	.008
Exposure to campaign	.302 [.091, .512]	.005	.545 [.372, 719]	< .001
Help seeking in past 12 months	− .222 [− .516, .072]	.138	− .168 [− .410, .074]	.174
Constant	4.957 [4.147, 5.496]	< .001	4.805 [4.359, 5.250]	< .001
R^2^	.033	< .001	.060	< .001

## Discussion

This study aimed to provide an updated evaluation of the awareness and effectiveness of ‘R U OK?Day’ by comparing campaign awareness and participation rates in 2017 to those reported in 2014, as well as investigating the impact of campaign exposure on intentions to help a close friend using recommended actions, beliefs about the importance, and ease, of asking are you okay?, and actual help provided to someone who might be experiencing personal difficulties.

### Summary and interpretation of findings

The findings are summarised and discussed in regard to the specific research questions they address.

### Has campaign awareness and participation in 2017 increased since 2014?

Both campaign awareness and participation were found to have significantly increased since 2014 [32]. Overall awareness rates increased by 12%, from 66% in 2014 to 78% in 2017, and overall participation rates increased by 13%, from 19% in 2014 to 32% in 2017. This indicates that campaign activities have been successful in continuing to raise awareness and encourage participation in R U OK?Day. The R U OK?Day campaign is unique in that it is a longer-term suicide prevention campaign with evaluation data collected annually, which therefore limits the comparisons that can be drawn to other campaigns.

### Has campaign awareness and participation across age groups increased in 2017 compared to 2014?

Significant increases in both campaign awareness and participation were observed in all age groups except for awareness in the 16–24 years group, possibly due to an already high awareness in 2014 amongst this age group. Large increases were observed in both campaign awareness and participation in the 65+ age group, meaning these rates are less disparate and are now comparable with the rates of the 55–64 age group. As campaign efforts specifically targeting this age group have not taken place, some of this increase could be attributable to some people who were previously in the 55–64 age group in 2014 having moved into the 65+ age group in 2017.

### Is there a significant difference in campaign awareness between those who sought help from a mental health professional in the past 12 months compared to those who didn’t?

Increased campaign awareness and participation was also found to be associated with seeking help from a mental health professional in the past 12 months, which suggests that campaign exposure may influence individuals to seek professional help for themselves. However, this finding could also suggest that individuals who have sought help from a mental health professional in the past 12 months are more likely to be exposed to the R U OK?Day campaign (i.e., more likely to receive online or social media advertising from related internet searches). Therefore, conclusions cannot be drawn about the directionality of this relationship. Other suicide prevention campaigns have found campaign exposure to be linked to increases in help seeking from a mental health professional and increases in calls to help lines [5]. Although not a direct campaign message, campaign exposure may have a positive consequence of improving individual help-seeking.

### Does campaign exposure predict intentions to help a close friend who might be, or is obviously, troubled about something, using recommended actions?

Campaign exposure (combined awareness and participation) was found to significantly predict intentions to use recommended actions to help a close friend who might be experiencing personal difficulties or is obviously experiencing personal difficulties. Campaign awareness was associated with participants being twice as likely to have intentions to use recommended actions to help a close friend who might be experiencing personal difficulties, with campaign participation extending this to being three times as likely to have intentions to help using recommended actions, compared to those with no exposure. Further, campaign awareness was associated with participants being almost three times as likely to have intentions to help a close friend who was obviously troubled using recommended actions compared to those with no awareness. Again, reverse causation (i.e., people with greater helping intentions being more likely to pay attention to the campaign) is possible, as is confounding by other factors such as exposure to people with mental health problems. Nevertheless, these findings suggest that the R U OK? campaign has been successful in influencing the Australian community’s belief that they should help a friend showing early signs of distress.

### Does campaign exposure predict stronger belief in the importance of asking, as well as ease of asking a close friend who appeared to be experiencing personal difficulties about what’s troubling them?

Having stronger beliefs about the importance of asking a friend who appeared to be experiencing personal difficulties about what’s troubling them and the ease of asking about their wellbeing was significantly associated with increased campaign exposure. Females had significantly greater helping beliefs, which is consistent with the research literature that shows that females are more likely to have higher empathy and provide support, and are more comfortable talking about emotions [40, 41].

### Does campaign exposure predict increased likelihood of reaching out to someone who might be experiencing personal difficulties?

Campaign exposure predicted helping behaviour. Specifically, participants with highest exposure (awareness and participation) were found to be six times more likely to reach out to someone who might be experiencing personal difficulties, compared to those with no exposure. The increase in helping behaviour and helping beliefs associated with campaign exposure suggests the R U OK?Day campaign is achieving its aims to empower and encourage reaching out to others to help someone who might be experiencing personal difficulties.

### Comparison to other research findings

The findings of the current evaluation are consistent with previous research where exposure to campaigns aiming to encourage helping conversations has been found to increase intentions of talking to someone who was experiencing personal difficulties [9, 10]. However, such health promotion campaigns are rare and limited research has been conducted on the impact of broader public health campaigns on providing actual support to others. This makes it difficult to compare the current findings as changes in behavioural outcomes from campaign exposure, such as help-seeking, have been more mixed compared to other outcomes [5]. Further quantitative and qualitative research into which elements and campaign messaging make a campaign successful, and the mechanism of campaign effects, are required.

The Theory of Planned Behaviour [42] offers an explanation of how beliefs in the importance of, and ease in, helping and helping intentions, all of which are influenced by the R U OK?Day campaign, combine to create meaningful behaviour change. Helping behaviour is influenced by intentions to help, which in turn are influenced by attitudes towards helping others and the perceived social norms around providing support, as well as perceived control and ability for the actions to be carried out. Exposure to the R U OK? campaign has been shown to influence helping attitudes and awareness of the importance of social support in preventing suicide. Furthermore, the campaign aims to encourage reaching out and improve ease of having a conversation with someone who might be experiencing personal difficulties by providing advice through a simple four-step model on how to have that conversation. The longer-term nature of R U OK?Day has enabled it to achieve community support and foster sufficient exposure to generate significant change [6].

### Strengths and Limitations

This study provides an updated evaluation of the impact of the R U OK?Day campaign. Ongoing evaluation is important to ensure that the campaign is having the intended impact, and for monitoring changes and/or any cumulative impacts over time. For example, the previous evaluation highlighted demographic groups that are lower in campaign exposure [32], such as the 65+ age-group, and our study showed that awareness in this group has improved over time. This study was also able to extend the findings of the past evaluation by reporting on intentions to help using recommended actions and behaviours due to the availability of these new data types as was recommended by the initial evaluation [32]. Further, the improved consistency of measures used in the annual surveys allowed us to compare 2017 data to that obtained in 2014.

There were also some limitations in this study. First, the actual impact of the campaign on suicide rates was not measured which therefore limits conclusions that can be drawn about the campaign’s ability to reduce these, particularly within a cross-sectional study design. Second, participants were selected through an online panel and therefore may not be representative of the Australian population, particularly parts of the community who are less technology literate, and those with an interest in mental health are potentially over-represented. Third, it is possible that 2014 survey respondents may have participated in the 2017 survey, which would be a minor violation of independence assumptions for chi-square analyses, although it is expected that the number of repeat respondents is low based on the panel from which the sample was drawn constantly changing. Fourth, participant interpretation of the term ‘troubled’ is subjective and may have influenced responding depending on individual interpretation. Another limitation is that the reliability and validity of the survey questions that were developed to specifically evaluate the R U OK?Day campaign are unknown, and therefore results should be interpreted with caution. Finally, knowledge of how to start a conversation with someone who might be experiencing personal difficulties using the four steps developed by R U OK?, as well as knowledge of suicide and mental health issues, were not measured in the current study. Including these measures could provide useful information to help shape and promote the campaign’s key messages going forward.

### Implications and recommendations for future research

Our findings suggest that public awareness campaigns that aim to improve social support can be effective in generating broad public awareness of, support for, and undertaking helping behaviours through positive helping conversations. R U OK? has been successful in increasing the awareness of its campaign message and is encouraged to continue its efforts to positively impact the Australian population. As the positive effects of the campaign are enhanced by participation, future campaigns should aim to increase participation in demographic groups who have reported the lowest rates by developing innovative ways to encourage each group. These target groups include the 55–64 and 65+ age groups, as well as males in general due to their lower reporting of awareness and helping behaviours compared to females. In moving forward and continuing to increase the positive impact of the campaign, developing targets in relation to awareness and participation rates may assist in increasing the momentum of the campaign, as being a public health promotion campaign, its effectiveness is related to the spread of total population reach.

R U OK?Day is a unique public health campaign given its longer-term duration as well as its annual evaluations, which increases its potential to impact reaching out to others and help-seeking behaviour compared to shorter once-off campaigns. It is important that the ongoing impact of the R U OK?Day campaign continues to be rigorously evaluated. Future evaluations could investigate the reactions of the recipients of the R U OK? helping conversations to better understand the impact of this support and assess community knowledge of the four-step model promoted as part of the key campaign messages. In addition, the annual surveys could elicit information about the campaign resources accessed and utilised so that future evaluation can determine whether these resources are improving knowledge on how to have conversations about personal difficulties.

## Conclusions

Our findings have shown that both awareness of, and participation in, the R U OK?Day campaign have continued to increase. They suggest that the R U OK?Day campaign is relevant and effective in spreading the message about the importance of reaching out to vulnerable others. The link between campaign exposure and helping beliefs, intentions and behaviours provides evidence that the campaign is successful in educating and empowering the Australian community to have conversations with someone who might be experiencing personal difficulties. It is vital that campaign evaluation is continued to monitor its ongoing impact and relevance.

## Data Availability

The data that support the findings of this study are owned by R U OK? Limited.
